# The Effect of the Targeted Recombinant Toxin DARPin-PE40 on the Dynamics of HER2-Positive Tumor Growth

**Published:** 2017

**Authors:** E.A. Sokolova, G.M. Proshkina, O.M. Kutova, I.V. Balalaeva, S.M. Deyev

**Affiliations:** Shemyakin–Ovchinnikov Institute of Bioorganic Chemistry, Russian Academy of Sciences, Miklukho-Maklaya Str., 16/10, Moscow, 117997, Russia; Lobachevsky State University of Nizhny Novgorod, Gagarin Ave., 23, Nizhny Novgorod, 603950, Russia; Lomonosov Moscow State University, Faculty of Biology, Leninskie Gory, 1, build. 12, Moscow, 119234 , Russia

**Keywords:** non-immunoglobulin module DARPin, Pseudomonas aeruginosa exotoxin A, HER2 receptor, targeted therapy

## Abstract

The development of targeted toxins based on non-immunoglobulin targeting molecules appears to be one of the most advanced approaches in the targeted therapy of malignant tumors with a high expression of the HER2 receptor. Earlier, we showed that the targeted toxin DARPin-PE40 consisting of the HER2-specific non-immunoglobulin polypeptide (the targeting module) and a fragment of *Pseudomonas *exotoxin A (the toxic module) exhibits an antitumor effect *in vivo *against the HER2-positive adenocarcinoma xenograft. In this work, an in-depth analysis of the effect of DARPin-PE40 on the growth dynamics of experimental xenograft tumors was carried out. DARPin-PE40 was shown to inhibit tumor growth at a dose of 25 and 50 μg/animal and to cause tumor node reduction at a dose of 80 μg/animal, followed by growth resumption at the end of therapy. An evaluation of the tumor growth dynamics revealed statistically significant differences in tumor volume in mice in the experimental groups compared to the control group. The results testify to the potential of using the created targeted toxin as an agent for the targeted therapy of HER2-overexpressing tumors.

## INTRODUCTION

According to the statistical data provided by P.A. Herzen Moscow Oncology Research Institute in 2015, breast cancer is the most common malignant disease affecting women, accounting for 20.9% of the total number of newly diagnosed neoplasms [1]. Breast cancer also sadly holds a leading place in cancer mortality among the female population of Russia, reaching 17% in 2015. As for the global statistics, about 1.6 million women are diagnosed with breast cancer every year, and about 500,000 die of the disease.

To sum up these facts, it is obvious that the development of novel antitumor agents and new approaches to cancer therapy is a priority. Targeted therapy has been developing rapidly in recent years. The approach consists in a targeted attack on tumor cells by using specific bifunctional therapeutic agents that are capable of selectively binding to tumor cells, on the one hand, and effectively eliminating them, on the other hand [2].

The HER2 receptor, which belongs to the human epidermal growth factor receptor family, is one of the best studied therapeutic targets [3, 4]. The tyrosine kinase receptor HER2 is normally present in all types of human epithelial tissues with a density of several thousand molecules per cell. Amplification of the HER2 gene under malignant cell transformation leads to overexpression of the receptor encoded. The HER2 receptor also becomes capable of constitutive heterodimerization with other receptors of the family (HER1, HER3, HER4). Continuous signal transmission from the membrane to the nucleus leads to an increase in cell proliferation, inhibition of apoptosis and, ultimately, tumor formation and metastasis. It is known that the level of HER2 gene expression is increased in 15-20% of human breast and ovarian cancers [3, 5].

Exotoxin A of *Pseudomonas aeruginosa *is one of the most effective protein toxins used in targeted therapy [6]. *Pseudomonas *exotoxin is a three-domain protein consisting of 613 a.a. We replaced the first domain of the exotoxin (1-252 a.a.), which is responsible for toxin binding to the natural receptor, with the HER2-specific non-immunoglobulin DARPin module [7], thus turning the exotoxin into a targeted toxin. A new generation of non-immunoglobulin targeting molecules based on artificial proteins with ankyrin repeats, DARPins, are increasingly used in molecular biology as targeting modules [8-10]. DARPins contain no cysteine residues, which allows for the production of these proteins directly in the *Escherichia coli *cytoplasm. They are also characterized by a high expression level in the bacterial system, monomeric state in solution with no tendency toward aggregation, and substantial resistance to proteases [11]. Because of these features, scaffold proteins have significant advantages over immunoglobulins as alternative targeting components of multifunctional compounds for the diagnosis and therapy of various diseases.

We analyzed the dynamics of the antitumor effect of the targeted toxin based on a fragment of *Pseudomonas aeruginosa *exotoxin A and the HER2-specific scaffold protein DARPin *in vivo *on the xenograft model of human breast adenocarcinoma with high expression of the target receptor HER2.

## EXPERIMENTAL

**Preparation of a highly purified targeted toxin, DARPin-PE40, for in vivo studies**

The DARPin-PE40 gene was expressed in *E. coli *strain BL21 (DE3) cells as described in [12]. Fresh transformants (one colony per ml) were introduced in 25 ml of the auto-induction medium TBP-5052 [13] containing 2 mM MgSO_4_, 25 mM Na_2_HPO_4_, 25 mM KH_2_PO_4_, 50 mM NH_4_Cl, 0.5% glycerol, 0.05% glucose, 0.2% lactose, 0.5% yeast extract, 1% tryptone, and 0.1 g/l ampicillin and grown in a 250 ml flask for 24 h at 25 °C until the culture density reached OD_600_ of 20-25. The cells were harvested by centrifugation on a cooled centrifuge at 6,000 *g *for 10 min. The pellet was re-suspended in 10 ml of lysis buffer (200 mM Tris-HCl, 500 mM sucrose, 1 mM EDTA, pH 8.0, 60 μg/ml lysozyme). The suspension was diluted with sterile water and incubated for 30 min at room temperature. The cells were then lysed on ice using a Vibra Cell sound disruptor (Sonics, USA) in a cycle mode of 10 s sonication, followed by 10 s cooling, for a total of 30 cycles. Cell debris was removed by centrifugation at 15,000 *g *for 20 min on a cooled centrifuge. The PMSF protease inhibitor (1 mM) and NaCl (100 mM) were added to the cleared supernatant. In order to remove the nucleic acids, polyethylenimine was added to the supernatant dropwise under constant stirring to a final concentration of 0.03%. The lysate was stirred for an additional 15 min at 4 °C and centrifuged at 15,000 *g *for 20 min. The resulting lysate was filtered through a 0.22 μm filter. Imidazole (30 mM final concentration) and NaCl (500 mM final concentration) were then added, and the solution was loaded to a Ni^2+^- NTA column (GE Healthcare, USA) equilibrated with buffer: 20 mM Na-Pi, pH 7.5, 500 mM NaCl, 30 mM imidazole). The DARPin-PE40 protein was eluted using a linear gradient of imidazole (30-500 mM). The fraction eluted at ~ 150 mM imidazole was used for purification using ion exchange chromatography. The buffer was exchanged with one containing 20 mM Tris-HCl, pH 8.0, 150 mM NaCl using a PD10 column (GE Healthcare, USA). The protein solution was diluted three times with 20 mM Tris-HCl, pH 8.0, and applied on a MonoQ5/50 GL column (GE Healthcare, USA) equilibrated with 20 mM Tris-HCl, pH 8.0. A linear gradient of NaCl (0-1 M) was used to elute the protein. DARPin- PE40 was eluted at a NaCl concentration of about 500 mM. The yield of the target protein was 140 mg per liter of culture.

**Evaluation of the antitumor efficacy of DARPin-PE40 in vivo**

The antitumor activity was determined using a human tumor xenograft. Six- to eight-week-old athymic BALB/c nude mice were subcutaneously inoculated with 107 cells of human breast adenocarcinoma SK-BR-3 in 200 μl of phosphate-buffered saline. HER2 overexpression in tumor tissue was confirmed *ex vivo *by immunohistochemical analysis using the HercepTest kit (DAKO, USA). Tumor growth was monitored by the standard method for determining tumor size by measuring two diameters using a caliper. The tumor volume was calculated using the equation: *V *= *a *× *b*2 / 2, where *a *represents a larger diameter; and *b*, a smaller diameter [14]. Starting on day 9 after tumor cell inoculation, when the average tumor volume was ~ 100 mm3, the animals were randomly divided into the experimental and control groups (five animals per group). Animals in the experimental groups received 200 μl intravenous injections of DARPin-PE40 in phosphate buffered saline daily at a total dose of 25 μg/animal (five injections of 5 μg on days 9, 11, 13, 15, and 17), 50 μg/animal (five injections of 10 μg on days 9, 11, 13, 15, and 17) or 80 μg/animal (four injections of 20 μg DARPin-PE40 on days 9, 11, 13, and 15). The animals in the control group received 200 μl of phosphate-buffered saline on days 9, 11, 13, 15, and 17 after tumor cell inoculation. When the tumor node reached a volume of ~ 2500 mm3, the animals were euthanized. To plot tumor growth curves, the calculated tumor volume values were used, expressed as a percentage of the values at the initial time point (on the therapy start day). The data were represented as the mean ± standard error of the mean at each time point.

To quantify the antitumor effect, the initial stage of the tumor growth curve in the animals of each group was fitted by the equation *V *= *V0 *× *ekt*, where *V0 *represents the tumor node volume at the initial time corresponding to therapy start, and *k *is the tumor growth rate coefficient.

The *k *values were determined by linearization of the exponential phase of tumor growth (i.e. taking the logarithm of the tumor volume), followed by linear approximation. The tumor doubling time was calculated using the equation ln2/*k*. The data were represented using the box-and-whiskers diagram reflecting the median, the 25^th^ and 75^th^ percentiles, and the spread of values in each animal group.

## RESULTS AND DISCUSSION

We have previously created the recombinant targeted toxin DARPin-PE40 and studied its properties *in vitro *as a targeted agent for the highly effective targeted therapy of HER2-positive tumors. This agent possesses an antitumor effect that comes to inhibiting the growth of xenograft tumors *in vivo *[15]. The targeting module in this construct consists of a molecule of non-immunoglobulin nature based on an artificial ankyrin repeat protein, DARPin, capable of recognizing the HER2 receptor with high affinity (KD = 3.8 nM) [7]. The PE40 fragment of *Pseudomonas *exotoxin A (M = 40 kDa), which lacks a natural receptor-binding domain, is used as a cytotoxic module [16]. The genetic construct encoding this fusion protein was expressed in *E.coli *BL21(DE3) cells. The DARPin-PE40 fusion protein was purified by metal-chelate affinity and anion exchange chromatography.

An in-depth analysis of the effect of DARPin-PE40 on the dynamics of experimental tumor growth *in vivo *was carried out. Athymic BALB/c nude mice (6-8 weeks old) with subcutaneously established human breast adenocarcinoma SK-BR-3 (see the Experimental section) were repeatedly injected intravenously with DARPin-PE40 at a total dose of 25, 50 or 80 μg per animal (1.25, 2.5 or 4 mg/kg, respectively).

**Fig. 1:**
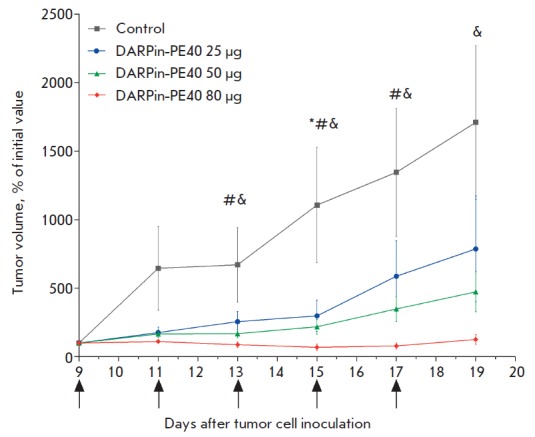
The curves of SK-BR-3 xenograft tumor growth in different groups of animals. The day of subcutaneous inoculation of SK-BR-3 cells to animals was set as day 0. The days of DARPin-PE40 injection are indicated with arrows. *, #, & – the statistically significant difference between the control and experimental groups: 25 μg, 50 μg and 80 μg DARPin-PE40, respectively (p < 0.05, Dunnett’s test, n = 5).

**Fig. 2:**
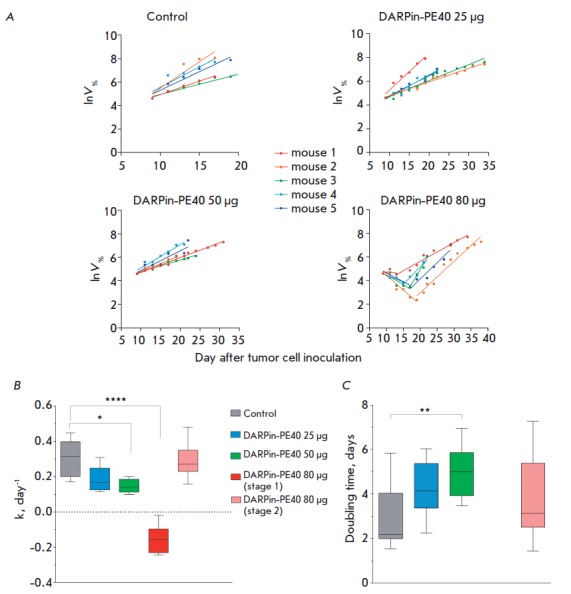
Analysis of SKBR- 3 xenograft tumor growth in different groups of animals. A – Linearization of the exponential phase of tumor growth (V% represents tumor volume as a percentage of that at the therapy start time). Data are shown for individual animals in each group. B – The box-and-whisker plot of the tumor growth rate coefficient (k). C – The box-and-whisker plot of the tumor doubling time (* – p < 0.05, ** – p < 0.01, **** – p < 0.0001).

The tumor growth dynamics showed a pronounced antitumor effect of the recombinant targeted toxin DARPin-PE40: statistically significant differences in the tumor volume in mice of the experimental groups were found in comparison with the control animals (p < 0.05) (*Fig. 1*). The tumors in control animals, as well as in the animals treated with 25 and 50 μg DARPin- PE40, exhibited an exponential growth at the initial stage (*Fig. 2A*). Meanwhile, the tumors grew significantly slower after DARPin-PE40 treatment: a statistically significant decrease in the tumor growth rate coefficient (*Fig. 2B*) and, correspondingly, an increase in the tumor doubling time (*Fig. 2C*) in the experimental groups compared with the control group were observed. This effect can be explained by a reduction in the pool of proliferating cancer cells in the growing tumor node as a result of the cytotoxic effect of the targeted toxin. Experimental tumors treated with a maximum dose of DARPin-PE40 (80 μg/animal) showed two distinct stages of growth. At stage 1, during the DARPin-PE40 injections, an exponential decrease in tumor volume on average by 60% relative to the volume registered at the beginning of the therapy was observed. At stage 2, after the DARPin-PE40 treatment was completed, the tumors resumed exponential growth (*Fig. 2*).

Taking into account tumor heterogeneity and the high genetic instability of tumor cells [17], the insufficient effectiveness of DARPin-PE40 may be caused by the presence or emergence of a resistant tumor cell population that leads to further tumor progression after the therapy is ended. In addition, since the experimental conditions simulated the situation of a therapeutic effect on an already formed tumor node, the limitations in the efficacy of the targeted toxin can also be related to its insufficient penetration into the tumor tissue. This, in turn, is due to a number of structural features of the tumor *in vivo*, including numerous cell-cell contacts, interstitial fluid pressure, and the presence of the extracellular matrix. Thus, along with an increase in dosage and/or therapy duration, the antitumor effect of the recombinant targeted toxin DARPin- PE40 could be enhanced by combining its action with a targeted increase in its permeability and accumulation in the tumor. This problem has been variously addressed; in particular, via the control of the formation of the extracellular matrix components and/or their degradation [18-20], as well as temporary disruption of cell-cell contacts in the tumor [21]. The latter approach proved effective with the use of HER2-specific full-length therapeutic antibodies [22] and, apparently, is one of the promising ways to develop targeted antitumor therapy.

## CONCLUSION

The use of non-immunoglobulin scaffold proteins, in particular DARPins, as targeting molecules is relevant for the development of new agents for targeted antitumor therapy. The dynamics of the antitumor activity of the targeted toxin DARPin-PE40, in which HER2-specific DARPin is fused with a toxic fragment of *Pseudomonas* exotoxin A into a single polypeptide chain, was studied. The effectiveness and reliability of the DARPin-PE40 antitumor effect demonstrate that it is a promising candidate for further study as an agent for the targeted therapy of tumors with high expression of the HER2 receptor.

## Abbreviations

DARPin: designed ankyrin repeat protein
EDTA: ethylenediaminetetraacetic acid
HER 1-4: human epidermal growth factor receptor 1-4
Ni2+-NTA: nickel nitrilotriacetic acid
PE40: fragment of Pseudomonas aeruginosa exotoxin A
PMSF: phenylmethylsulfonyl fluoride
a.a.: amino acid
